# Electronic Cigarette Use and Other Factors Associated with Cigarette Smoking among Thai Undergraduate Students

**DOI:** 10.3390/healthcare10020240

**Published:** 2022-01-26

**Authors:** Phantara Chulasai, Surarong Chinwong, Purida Vientong, John J. Hall, Dujrudee Chinwong

**Affiliations:** 1 PhD’s Degree Program in Pharmacy, Faculty of Pharmacy, Chiang Mai University, Chiang Mai 50200, Thailand; panthara.chula@gmail.com; 2 Department of Social Pharmacy, Faculty of Pharmacy, Payap University, Chiang Mai 50000, Thailand; 3 Department of Pharmaceutical Care, Faculty of Pharmacy, Chiang Mai University, Chiang Mai 50200, Thailand; surarong@gmail.com (S.C.); purida.v@cmu.ac.th (P.V.); 4 Center of Excellence for Innovation in Analytical Science and Technology for Biodiversity-Based Economic and Society (I-ANALY-S-T_B.BES-CMU), Chiang Mai University, Chiang Mai 50200, Thailand; 5 School of Population Health, Faculty of Medicine, University of New South Wales, Sydney 2052, Australia; john.hall@unsw.edu.au

**Keywords:** cigarette, cigarette smoking, electronic cigarette, e-cigarette, e-cigarette use, undergraduate student

## Abstract

The prevalence of smoking among young adults in Thailand has gradually increased. Therefore, this study aimed to identify factors associated with cigarette smoking among undergraduate students. This cross-sectional study used a self-administered, anonymous online questionnaire to gather data from undergraduate students across four universities in Chiang Mai Province, Thailand. All 1126 participants were an average age of 21.30 years old (SD 1.48). The findings revealed seven factors significantly associated with cigarette smoking (*p* < 0.05), including male sex, having no medical conditions, consuming alcohol daily and consuming alcohol in the past, having brothers or sisters who smoked cigarettes, having a father or mother who smoked cigarettes, having parents who considered smoking acceptable and having parents who had uncertain concerns about smoking, and had or have used electronic cigarettes (e-cigarettes). These associated factors could be useful in implementing appropriate tobacco-control programs to prevent cigarette smoking among undergraduate students. Relevant organizations, universities and healthcare professionals should communicate correct and appropriate information about the illness and diseases caused by using tobacco products to strengthen the correct perceptions of the harms of cigarette smoking and e-cigarette use among undergraduate students. Furthermore, smoke-free policies should be monitored and strictly enforced, particularly in university areas.

## 1. Introduction

Tobacco use is among the leading causes of global diseases, accounting for 6.4 million deaths yearly [1]. In Thailand, tobacco smoking remains a risk factor considered a significant cause of attributable death; 11.2% of the national deaths are ascribed to tobacco smoking [2]. Additionally, economic loss from the tobacco-related burden accounted for 0.78% of the national gross domestic product and 18.19% of the total health expenditure [3].

Since the WHO Framework Convention on Tobacco Control (WHO FCTC) presented MPOWER, a package of six key measures to assist nations in reducing tobacco demand, the global tobacco smoking rate has decreased from 22.7% in 2007 to 17.5% in 2019 [4]. Thailand has been acknowledged as a country with substantial success in tobacco control, complying with the WHO FCTC [5]. The prevalence of smoking across all age groups has continually declined from 21.2% in 2007 to 19.1% in 2017 [6]. Several tobacco control regulations have been enacted to strengthen the decrease of young adult smoking. For example, the legal purchasing age for tobacco products has been raised from 18 to 20 years, and smoking is now prohibited in all academic institutions or places of education and training [5,7]. However, challenges remain in reducing the gradual increase in the prevalence of smoking among young adults, from 19.9% in 2013 to 20.4% in 2017 [6]. Furthermore, in recent years, increases in electronic nicotine-delivery systems (ENDS) use have proliferated worldwide, especially in younger populations. In 2021, the WHO estimated the prevalence of ENDS ever-use among children and adolescents in all countries to be 19.9%, with 8.8% of current use (use in the last 30 days) [4]. One of the most common ENDS is the electronic cigarette (e-cigarette). In 2020, 18.1% of Thai university students reported using e-cigarettes [8]. This suggested that tobacco-control measures for young adult populations must be more comprehensive and practical.

Young adulthood is a developmental period critical to establishing risky health behaviors that are ongoing through adulthood [9]. Most smokers initiate their cigarette smoking before adulthood [10]; approximately one-third to one-half started smoking when they were university students [11,12,13]. Findings from a national survey in Thailand demonstrated that daily smokers experienced their first smoking at an average age of 18.1 years [6]. Therefore, preventing cigarette smoking is just as important as promoting smoking cessation in young adult populations. To accomplish this, information about the factors associated with cigarette smoking among young adults must be determined.

Despite the importance of understanding factors associated with cigarette smoking among young adults, prior studies in Thailand were limited to a specific group of students from each public university [12,13,14,15]. This study was conducted to obtain data from students at various public and private universities and aimed to identify factors associated with cigarette smoking among undergraduate students. The finding could pave the way to develop effective tobacco-control programs for undergraduate students.

## 2. Materials and Methods

### 2.1. Study Design and Participants

This cross-sectional study included university students at the undergraduate level from four universities, three public universities, and one private university in Chiang Mai Province, northern Thailand. Participants comprised undergraduate students 18 years or older, able to complete the online questionnaire on the Google Forms platform, able to communicate in Thai, and willing to join this study. They were recruited by convenience sampling method and snowball sampling technique: participants told friends to join this study. Recruitment was promoted using social media advertising, including posting on Facebook, Twitter, and Line user pages. This study provided no compensation for participants.

### 2.2. Sample Size Calculation

The study sample size was generated using a quota on cigarette-smoking status to increase the participation of undergraduate students who were cigarette smokers. The participants were weighed in a 1:1 ratio of cigarette smokers to noncigarette smokers. Then the study sample size was computed based on Yamane’s formula to determine sample size for a finite population with the following considerations [16]: the number of undergraduate students enrolled in four universities at the time this study was conducted, in the academic year 2018, totaled 68,105 [17], and the prevalence of smoking was 20.4% [6], suggesting 13,893 were smokers, and a margin of error was set at 0.05. Therefore, the sample size for this study comprised 389 participants who were cigarette smokers and 389 participants who were noncigarette smokers.

### 2.3. Questionnaire Development

The questionnaire was deliberately developed after a review of relevant studies in the related literature. Three experts in the field of smoking behaviors assessed the content validity of the questionnaire items to determine whether they met all of the study’s objectives. Following that, the Index of Item-Objective Congruence (IOC) was identified. Items with IOC scores greater than or equal to 0.5 were deemed appropriate; those with IOC scores less than 0.5 were deemed inappropriate and were revised in compliance with expert recommendations. The paper-based questionnaire was piloted by 31 undergraduate students uninvolved in the study to assess the use of appropriate language. The modified questionnaire was then converted to an online version using the Google Forms platform and was verified before being used in this study.

### 2.4. Data Collection

Data were collected using a self-administered, anonymous online questionnaire. Individuals interested in participating in the study could access the online questionnaire by clicking on the hyperlink or scanning the questionnaire QR code. Potential participants obtained a brief study description, including inclusion criteria, to determine their eligibility using an electronic subject information sheet. Researchers’ contact information was readily available for the participants to ask any questions before completing the questionnaire. Participants were informed that they were able to quit at any time without providing a reason and that all information would be anonymous and kept strictly confidential. The online questionnaire began after eligible participants confirmed their agreement to participate by approving the online informed consent. The online questionnaire required participants to answer each question and proceed to the end of the online questionnaire (approximately 15 min). Participants were encouraged to share the questionnaire hyperlink or QR code with their contacts and online platforms.

### 2.5. Measures

Data gained from participants comprised the following subsets of measures:

#### 2.5.1. Cigarette-Smoking Status

Cigarette-smoking status was the outcome variable of this study. Participants were defined as cigarette smokers or noncigarette smokers according to their self-report by responding to the question, “How would you identify your cigarette-smoking behavior”? When they chose “smoking”, they were classified as cigarette smokers. However, when they chose “never” or “used to smoke”, they were classified as noncigarette smokers.

#### 2.5.2. Sociodemographic and Smoking-Related Factors

Sociodemographic factors were assessed regarding sex (female or male), age (years), monthly income (≤10,000 or >10,000 THB), accommodation (with parents, on-campus housing, or off-campus housing), residing with others (alone, friends, boyfriend/girlfriend, parents, or relatives), medical conditions (yes or no), and alcohol consumption (never consume, consume every day, used to consume, or occasionally consume).

Smoking-related factors were assessed regarding peer smoking status: friends; relatives; boyfriend or girlfriend; brothers or sisters; and father or mother (yes or no), parental perception of cigarette smoking (unacceptable, acceptable, uncertain, or without comment), and overall opinion about cigarette smoking (positive, neutral, or negative). Participants were defined as having been e-cigarette users or never having been e-cigarette users by responding to the question, “How would you identify your e-cigarette use behavior?” When the participants chose “using” or “used to use”, they were classified as e-cigarette users. However, when they chose “never used”, they were classified as never having been e-cigarette users.

#### 2.5.3. Cigarette-Smoking Behaviors

Cigarette-smoking behaviors were assessed regarding age at cigarette-smoking initiation (years), the reason for first cigarette smoking (stress relief, peer pressure from friends, in social situations, self-curiosity, or imitating elders), and daily cigarette smoker (yes or no). Nicotine-dependence level was assessed using the Heaviness of Smoking Index (HSI) score. The overall HSI score ranged between zero and six (0 to 2 scores: low nicotine dependence; 3 to 4 scores: moderate nicotine dependence; and 5 to 6 scores: high nicotine dependence). These scores were summarized from answers to two questions, including daily cigarette consumption (1 to 10 cigarettes: 0 score; 11 to 20 cigarettes: 1 score; 21 to 30 cigarettes: 2 scores; and ≥31 cigarettes: 3 scores) and time to first cigarette of the day (≤5 min: 3 scores; 6 to 30 min: 2 scores; 31 to 60 min: 1 score; to ≥61 min: 0 score) [18].

### 2.6. Statistical Analysis

The main variables showed no missing data. STATA Software, Version 14 (StataCorp LP, College Station, TX, USA) was used to analyze the data with the significance level set as two-tailed with *p* < 0.05. Descriptive statistics for categorical variables was summarized as frequency and percentage, while continuous variables were summarized as means and standard deviation (SD). Inferential statistics, Fisher’s exact test, was used to assess the difference between two independent groups (cigarette smokers and noncigarette smokers) for categorical variables. The independent *t*-test was used for continuous variables. Factors associated with cigarette smoking among undergraduate students were determined using binary logistic regression analysis. The odds ratios (ORs) and 95% confidence intervals (95% CI) were used to calculate the associations. Univariable logistic regression was firstly performed to estimate OR. Independent variables found to be associated in univariable logistic regression (*p* < 0.05) were then entered in a multivariable model. Independent variables with a variance inflation factor (VIF) value > 2 were excluded. The final model from multivariable logistic regression analysis showed that multicollinearity among independent variables was not a cause for concern [19]. Furthermore, the goodness of fit for the final model was carried out using the Hosmer–Lemeshow test, with *p* ≥ 0.05 considered a good fit.

## 3. Results

### 3.1. General Characteristics of the Participants

In all, 1126 participants, including 494 cigarette smokers and 632 noncigarette smokers, completed the online questionnaire between December 2018 and February 2019. The majority of participants were female (56.2%; *n* = 633) with an average age of 21.30 years (SD 1.48). About one half of the participants consumed alcohol daily (50.3%; *n* = 566) and used e-cigarettes (49.8%; *n* = 561). Among 561 e-cigarette users, 490 were current e-cigarette users (data not shown). Cigarette smokers and noncigarette smokers statistically differed (*p* < 0.05) regarding their sociodemographic and smoking-related characteristics (Table 1).

### 3.2. Cigarette-Smoking Behaviors

The 494 cigarette smokers reported they began smoking cigarettes at an average age of 15.13 years (SD 2.46). The three most common reasons reported for first smoking cigarettes were smoking for stress relief (37.0%; *n* = 183), peer pressure from friends (26.5%; *n* = 131), and smoking in social situations (20.8%; *n* = 103). Almost all reported daily cigarette smoking (92.9%; *n* = 459), and the majority expressed moderate levels of nicotine dependence as evaluated by HSI score (66.2%; *n* = 327) (Table 2).

### 3.3. Factors Associated with Cigarette Smoking

Using multivariable logistic regression analysis, seven factors were found to be significantly associated with cigarette smoking: sex, medical conditions, alcohol consumption, brother’s or sister’s cigarette smoking, father’s or mother’s cigarette smoking, parental perception of cigarette smoking, and e-cigarette use. Male undergraduate students were more likely to smoke cigarettes than females. Furthermore, undergraduate students without medical conditions were more likely to smoke cigarettes than those who had. Undergraduate students who consumed alcohol daily and used to consume alcohol had a higher likelihood of cigarette smoking than those who had no consumption at all. Likewise, undergraduate students whose brothers or sisters smoked cigarettes and those whose father or mother smoked cigarettes were more likely to smoke cigarettes than those whose parents did not. In addition, undergraduate students whose parents considered that cigarette smoking was acceptable and those who were uncertain about their parents’ concerns on cigarette smoking had a higher likelihood of cigarette smoking than those whose parents considered that cigarette smoking was unacceptable. Furthermore, undergraduate students who had or have used e-cigarettes were more likely to smoke cigarettes than those who had not (Table 3).

Interestingly, when e-cigarette use was classified as current e-cigarette users (*n* = 490) or noncurrent e-cigarette users (*n* = 636), three factors were found to be significantly associated with cigarette smoking, including alcohol consumption, parental perception of cigarette smoking, and e-cigarette use (Table S1).

## 4. Discussion

### 4.1. Principal Findings

This study revealed that sex, medical conditions, alcohol consumption, brother’s or sister’s cigarette smoking, father’s or mother’s cigarette smoking, parental perception of cigarette smoking, and e-cigarette use were significantly associated with cigarette smoking among undergraduate students.

According to the associated factors, male undergraduate students were more likely to smoke cigarettes. This finding was consistent with related studies conducted in Korea [20] and New Zealand [21] concerning factors associated with smoking in the same target population. This could be explained with reference to sociocultural beliefs and social norms that cigarette smoking among females is considered an unacceptable behavior in Thai society [14]. Another possibility might be that male undergraduate students were more likely to have many smoking friends and experienced more independence from their families [21]. Moreover, undergraduate students without medical conditions were more likely to smoke cigarettes than those who had. The findings suggested that undergraduate students without medical conditions may be less aware of the health harms of cigarette smoking.

Additionally, undergraduate students who consumed alcohol daily and used to consume alcohol had a higher likelihood of cigarette smoking. This finding was consistent with related studies conducted in Korea [20]. The findings implied that the smoking behaviors of undergraduate smokers may be influenced by behavioral and social factors, particularly the social situation in which alcohol was consumed. This was strengthened by the findings of this study (Table 2) and another related study in Thailand [13], revealing that the common reasons for cigarette smoking among undergraduate smokers were to relieve stress, peer pressure from friends, and in social situations. Therefore, smoke-free policies in places where undergraduate students gather should be monitored and strictly enforced in this regard, not only in university areas but also restaurants, bars, clubs, nightclubs, coffee shops, and internet cafes.

In addition, undergraduate students whose family members smoked cigarettes, including their father, mother, brothers, or sisters, were more likely to smoke cigarettes. This finding was consistent with related studies conducted in Korea [20]. Environmental factors, such as the social influence of family members, are important determinants of cigarette smoking. Undergraduate students might adopt the attitude that cigarette smoking is acceptable from cigarette-smoking behaviors of their family members [15].

Furthermore, undergraduate students whose parents considered cigarette smoking acceptable and those uncertain about their parents’ concerns of cigarette smoking had a higher likelihood of cigarette smoking. This might be partly attributable to the fact that parental perception of cigarette smoking could influence undergraduate students’ decisions about whether to smoke cigarettes [22]. Those whose parents considered cigarette smoking to be unacceptable tended to avoid smoking and practiced self-restraint [22]. Strengthening the correct perception about cigarette smoking among undergraduate students as well as their parents would be essential to protect undergraduate students from initiating smoking behavior.

Our findings highlighted that those undergraduate students who had or have used e-cigarettes were more likely to smoke cigarettes. The findings were consistent with those discovered among young adult smokers in Korea [20]. Although many of the long term health implications of e-cigarette use remain uncertain, substantial evidence suggests that e-cigarettes pose dangers [4]. Increases in e-cigarette use have proliferated worldwide, especially in younger populations, because of the appeal of these products and promotional strategies [4]. Therefore, future studies should place importance on e-cigarettes use as well as dual use of e-cigarettes and cigarettes among undergraduate students. Furthermore, educational campaigns communicating correct information about e-cigarettes should be made a high priority.

This study constituted one of the few studies in Thailand that collected data from various public and private university students to identify factors associated with cigarette smoking. The findings appeared to be consistent with related studies conducted in other countries concerning the same target population and could be useful in implementing appropriate tobacco control programs to prevent cigarette smoking among undergraduate students. Relevant organizations, universities, and healthcare professionals should communicate correct and appropriate information about the illness and diseases caused by using tobacco products to strengthen the correct perceptions of the harms of cigarette smoking and e-cigarette use. Smoke-free policies in places where undergraduate students gather should be monitored and strictly enforced, particularly in university areas. Furthermore, our findings supported the need for future studies, which should investigate e-cigarette-use behaviors among undergraduate students.

### 4.2. Limitations

Several potential limitations were encountered in this study and should be considered in the interpretation of its findings. First, the data reported in this study, including smoking-related information and cigarette-smoking behaviors, were self-reported, which could constitute recall bias. Second, this study classified cigarette-smoking status as cigarette smoker and noncigarette smoker. Undergraduate students who used to smoke cigarettes were classified as being noncigarette smokers. The findings may limit comparing with other studies classifying cigarette-smoking status differently. Third, cigarette smoking is not well accepted in Thai society. Cigarette-smoking behaviors reported in this study could have been underreported according to sociocultural beliefs and social norms. Fourth, as this was a cross-sectional study, the researcher was unable to determine the causes and effects. Therefore, the findings could not indicate whether factors associated with cigarette smoking were present before cigarette-smoking initiation. Fifth, this study revealed seven factors significantly associated with cigarette smoking, using a multivariable logistic regression model. However, the findings of such a model have a limitation in assessing the true odds ratio of certain variables, as their strength of association with the outcome might have been affected by some other factors in the model. Further explanatory studies should be performed to evaluate the association between cigarette smoking and the potential factors found in this study. Finally, this study gathered information from participants recruited by convenience sampling method and snowball sampling technique. As a result, the findings may not be generalizable to all Thai undergraduate students.

## 5. Conclusions

This study contributed important knowledge about factors associated with cigarette smoking among undergraduate students in Thailand. The findings indicated that male sex, having no medical conditions, consuming alcohol daily and consuming alcohol in the past, having brothers or sisters who smoked cigarettes, having a father or mother who smoked cigarettes, having parents who considered smoking acceptable and having parents who had uncertain concerns about smoking, and had or have used e-cigarettes were significantly associated with cigarette smoking. These factors could be considered in implementing tobacco-control programs for undergraduate students.

## Figures and Tables

**Table 1:** General characteristics of the 1126 participants.

Characteristic	All Participants (*n* = 1126), *n* (%)	Cigarette Smokers (*n* = 494), *n* (%)	Noncigarette Smokers (*n* = 632), *n* (%)	*p*
Sex				
Female	633 (56.2)	242 (49.0)	391 (61.9)	<0.001
Male	493 (43.8)	252 (51.0)	241 (38.1)	
Age (years), mean (SD)	21.30 (1.48)	21.40 (1.20)	21.22 (1.66)	0.031
Monthly income (THB) ^1^				
≤10,000	574 (51.0)	233 (47.2)	341 (54.0)	0.026
>10,000	552 (49.0)	261 (52.8)	291 (46.0)	
Accommodation				
With parents	149 (13.2)	57 (11.5)	92 (14.6)	<0.001
On-campus housing	83 (7.4)	17 (3.4)	66 (10.4)	
Off-campus housing	894 (79.4)	420 (85.0)	474 (75.0)	
Residing with others				
Alone	299 (26.6)	97 (19.6)	202 (32.0)	<0.001
Friends	410 (36.4)	195 (39.5)	215 (34.0)	
Boyfriend/girlfriend	269 (23.9)	146 (29.6)	123 (19.5)	
Parents	111 (9.9)	38 (7.7)	73 (11.6)	
Relatives	37 (3.3)	18 (3.6)	19 (3.0)	
Medical conditions				
Yes	84 (7.5)	12 (2.4)	72 (11.4)	<0.001
No	1042 (92.5)	482 (97.6)	560 (88.6)	
Alcohol consumption				
Never	108 (9.6)	8 (1.6)	100 (15.8)	<0.001
Every day	566 (50.3)	335 (67.8)	231 (36.6)	
Used to	55 (4.9)	22 (4.4)	33 (5.2)	
Occasionally	397 (35.3)	129 (26.1)	268 (42.4)	
Friend’s cigarette smoking				
No	391 (34.7)	120 (24.3)	271 (42.9)	<0.001
Yes	735 (65.3)	374 (75.7)	361 (57.1)	
Relative’s cigarette smoking				
No	676 (60.0)	260 (52.6)	416 (65.8)	<0.001
Yes	450 (40.0)	234 (47.4)	216 (34.2)	
Boyfriend’s/girlfriend’s cigarette smoking				
No	724 (64.3)	288 (58.3)	436 (69.0)	<0.001
Yes	402 (35.7)	206 (41.7)	196 (31.0)	
Brother’s/sister’s cigarette smoking				
No	795 (70.6)	301 (60.9)	494 (78.2)	<0.001
Yes	331 (29.4)	193 (39.1)	138 (21.8)	
Father’s/mother’s cigarette smoking				
No	864 (76.7)	342 (69.2)	522 (82.6)	<0.001
Yes	262 (23.3)	152 (30.8)	110 (17.4)	
Parental perception of cigarette smoking				
Unacceptable	346 (30.7)	64 (13.0)	282 (44.6)	<0.001
Acceptable	482 (42.8)	282 (57.1)	200 (31.6)	
Uncertain	216 (19.2)	107 (21.7)	109 (17.2)	
Without comment	82 (7.3)	41 (8.3)	41 (6.5)	
Overall opinion about cigarette smoking				
Negative	479 (42.5)	141 (28.5)	338 (53.5)	<0.001
Neutral	627 (55.7)	344 (69.6)	283 (44.8)	
Positive	20 (1.8)	9 (1.8)	11 (1.7)	
Electronic cigarettes use				
Never	565 (50.2)	80 (16.2)	485 (76.7)	<0.001
Used	561 (49.8)	414 (83.8)	147 (23.3)	

Percentages may not total 100 because of rounding off; SD, standard deviation. THB, Thai baht; ^1^ 1 USD, 32 THB.

**Table 2:** Cigarette-smoking behaviors of the 494 cigarette smokers.

Smoking Behavior	*n* (%)
Age at cigarette-smoking initiation (years), mean (SD)	15.13 (2.46)
Reason for first cigarette smoking	
Stress relief	183 (37.0)
Peer pressure from friends	131 (26.5)
In social situations	103 (20.8)
Self-curiosity	74 (15.0)
Imitating elders	3 (0.6)
Daily cigarette smoker	
No	35 (7.1)
Yes	459 (92.9)
Daily cigarette consumption	
1–10	248 (50.2)
11–20	206 (41.7)
21–30	34 (6.9)
≥31	6 (1.2)
Time to first cigarette of the day	
≤5 min	269 (54.4)
6–30 min	146 (29.6)
31–60 min	57 (11.5)
≥61 min	22 (4.4)
Nicotine-dependence level (Heaviness of Smoking Index score) ^1^	
Low nicotine dependence (0–2 scores)	141 (28.5)
Moderate nicotine dependence (3–4 scores)	327 (66.2)
High nicotine dependence (5–6 scores)	26 (5.3)

Percentages may not total 100 because of rounding off; SD, standard deviation. ^1^ The scores ranged between 0 and 6, with 6 indicating the highest nicotine dependence level.

**Table 3:** Univariable and multivariable logistic regression analysis of factors associated with cigarette smoking among undergraduate students.

Factor	Crude OR (95%CI)	*p*	Adjusted OR (95%CI)	*p*
Sex				
Female	1.00		1.00	
Male	1.69 (1.33–2.14)	<0.001	1.49 (1.06–2.09)	0.021
Age	1.09 (1.004–1.18)	0.039	1.08 (0.95–1.22)	0.224
Monthly income (THB) ^1^				
≤10,000	1.00		1.00	
>10,000	1.31 (1.04–1.66)	0.024	0.87 (0.63–1.22)	0.428
Accommodation				
With parents	1.00		1.00	
On-campus housing	0.42 (0.22–0.78)	0.006	0.99 (0.39–2.50)	0.983
Off-campus housing	1.43 (1.00–2.04)	0.049	1.21 (0.65–2.24)	0.548
Residing with others				
Alone	1.00		1.00	
Friends	1.89 (1.38–2.58)	<0.001	1.33 (0.85–2.07)	0.211
Boyfriend/girlfriend	2.47 (1.76–3.48)	<0.001	1.47 (0.92–2.35)	0.108
Parents	1.08 (0.68–1.72)	0.731	1.41 (0.66–3.03)	0.380
Relatives	1.97 (0.99–3.93)	0.053	1.11 (0.44–2.84)	0.821
Medical conditions				
Yes	1.00		1.00	
No	5.16 (2.77–9.63)	<0.001	2.43 (1.10–5.39)	0.028
Alcohol consumption				
Never	1.00		1.00	
Every day	18.13 (8.65–37.97)	<0.001	7.37 (2.97–18.28)	<0.001
Used to	8.33 (3.39–20.49)	<0.001	4.24 (1.38–13.02)	0.012
Occasionally	6.02 (2.84–12.74)	<0.001	2.30 (0.95–5.59)	0.066
Boyfriend’s/girlfriend’s cigarette smoking				
No	1.00		1.00	
Yes	1.59 (1.24–2.03)	<0.001	0.95 (0.66–1.37)	0.779
Brother’s/sister’s cigarette smoking				
No	1.00		1.00	
Yes	2.30 (1.77–2.98)	<0.001	1.58 (1.08–2.29)	0.017
Father’s/mother’s cigarette smoking				
No	1.00		1.00	
Yes	2.11 (1.59–2.79)	<0.001	1.51 (1.01–2.26)	0.045
Parental perception of cigarette smoking				
Unacceptable	1.00		1.00	
Acceptable	6.21 (4.48–8.61)	<0.001	2.72 (1.70–4.34)	<0.001
Uncertain	4.32 (2.96–6.33)	<0.001	2.72 (1.63–4.56)	<0.001
Without comment	4.41 (2.64–7.34)	<0.001	1.39 (0.70–2.76)	0.345
Overall opinion about cigarette smoking				
Negative	1.00		1.00	
Neutral	2.91 (2.26–3.75)	<0.001	1.16 (0.79–1.70)	0.445
Positive	1.96 (0.80–4.84)	0.144	1.88 (0.59–6.02)	0.286
Electronic cigarettes use				
Never	1.00		1.00	
Used	17.07 (12.62–23.10)	<0.001	18.87 (13.18–27.02)	<0.001

OR, odds ratio; CI, confidence intervals; THB, Thai baht; ^1^ 1 USD, 32 THB.

## Data Availability

The data presented in this study are available from the corresponding author on reasonable request.

## Supplementary Materials

The following are available online at https://www.mdpi.com/article/10.3390/healthcare10020240/s1, Table S1: Univariable and multivariable logistic regression analysis of factors associated with cigarette smoking among undergraduate students (classified e-cigarette use as current e-cigarette users (*n* = 490) or noncurrent e-cigarette users (*n* = 636)).
