# Supplementary material for: Electronic Cigarette Use and Other Factors Associated with Cigarette Smoking among Thai Undergraduate Students
Source: Healthcare (Basel). 2022 Jan 26;10(2):240. doi: 10.3390/healthcare10020240 (PMC8871931; doi:10.3390/healthcare10020240)
Supplement: Supplementary file 1 [file healthcare-10-00240-s001.zip › healthcare-1479898-supplementary.pdf]

healthcare

MDPI

Table S1. Univariable and multivariable logistic regression analysis of factors associated with cigarette smoking among undergraduate students (classified e-cigarette use as current e-cigarette users (n = 490) or noncurrent e-cigarette users (n = 636)).

Factor	Crude OR (95%CI)	*p*	Adjusted OR (95%CI)	*p*
Sex				
Female	1.00		1.00	
Male	1.69 (1.33–2.14)	<0.001	1.39 (0.96–2.01)	0.083
Age (years), mean (SD)	1.09 (1.004-1.18)	0.039	1.05 (0.91-1.20)	0.510
Monthly income (THB) [1]				
≤10,000	1.00		1.00	
>10,000	1.31 (1.04–1.66)	0.024	0.84 (0.59–1.21)	0.360
Accommodation				
With parents	1.00		1.00	
On campus housing	0.42 (0.22–0.78)	0.006	1.00 (0.35–2.80)	0.994
Off campus housing	1.43 (1.00–2.04)	0.049	1.23 (0.62–2.44)	0.550
Living with others				
Alone	1.00		1.00	
Friends	1.89 (1.38–2.58)	<0.001	1.60 (0.98–2.63)	0.061
Boyfriend/girlfriend	2.47 (1.76–3.48)	<0.001	1.60 (0.96–2.68)	0.071
Parents	1.08 (0.68–1.72)	0.731	1.22 (0.53–2.84)	0.637
Relatives	1.97 (0.99–3.93)	0.053	0.98 (0.36–2.70)	0.975
Medical conditions				
Yes	1.00		1.00	
No	5.16 (2.77–9.63)	<0.001	2.23 (0.93–5.35)	0.072
Alcohol consumption				
Never	1.00		1.00	
Every day	18.13 (8.65–37.97)	<0.001	6.10 (2.28–16.37)	<0.001
Used to	8.33 (3.39–20.49)	<0.001	3.28 (0.96–11.15)	0.058
Occasionally	6.02 (2.84–12.74)	<0.001	1.70 (0.64–4.51)	0.284
Boyfriend's/girlfriend's cigarette smoking				
No	1.00		1.00	
Yes	1.59 (1.24–2.03)	<0.001	0.83 (0.56–1.23)	0.357
Brother's/sister's cigarette smoking				
No	1.00		1.00	
Yes	2.30 (1.77–2.98)	<0.001	1.44 (0.96–2.16)	0.074
Father's/mother's cigarette smoking				
No	1.00		1.00	
Yes	2.11 (1.59–2.79)	<0.001	1.54 (0.99–2.40)	0.053
Parental perception of cigarette smoking				
Unacceptable	1.00		1.00	
Acceptable	6.21 (4.48–8.61)	<0.001	2.35 (1.40–3.93)	0.001
Uncertain	4.32 (2.96–6.33)	<0.001	2.21 (1.26–3.90)	0.006
Without comment	4.41 (2.64–7.34)	<0.001	0.88 (0.41–1.88)	0.740
Overall opinion about cigarette smoking				
Negative	1.00		1.00	
Neutral	2.91 (2.26–3.75)	<0.001	1.16 (0.76–1.76)	0.486
Positive	1.96 (0.80–4.84)	0.144	2.14 (0.64–7.16)	0.215
Current electronic cigarettes use				
No	1.00		1.00	

Yes	32.73 (23.53–45.53)	<0.001	36.45 (24.42–54.41)	<0.001

OR, odds ratio; CI, confidence intervals; THB, Thai baht; [1] 1 USD, 32 THB.
